# Dimension Reduction of Digital Image Descriptors in Neural Identification of Damaged Malting Barley Grains

**DOI:** 10.3390/s22176578

**Published:** 2022-08-31

**Authors:** Piotr Boniecki, Agnieszka Sujak, Agnieszka A. Pilarska, Hanna Piekarska-Boniecka, Agnieszka Wawrzyniak, Barbara Raba

**Affiliations:** 1 Department of Biosystems Engineering, Poznań University of Life Sciences, 50 Wojska Polskiego Str., 60-627 Poznań, Poland; 2 Department of Hydraulic and Sanitary Engineering, Poznań University of Life Sciences, 94A Piątkowska Str., 60-649 Poznań, Poland; 3 Department of Entomology and Environmental Protection, Poznań University of Life Sciences, 159 Dąbrowskiego Str., 60-594 Poznań, Poland

**Keywords:** digital image, graphic descriptors, PCA (principal component analysis), compression of graphical data, classification of quality, malting barley

## Abstract

The paper covers the problem of determination of defects and contamination in malting barley grains. The analysis of the problem indicated that although several attempts have been made, there are still no effective methods of identification of the quality of barley grains, such as the use of information technology, including intelligent sensors (currently, quality assessment of grain is performed manually). The aim of the study was the construction of a reduced set of the most important graphic descriptors from machine-collected digital images, important in the process of neural evaluation of the quality of BOJOS variety malting barley. Grains were sorted into three size fractions and seed images were collected. As a large number of graphic descriptors implied difficulties in the development and operation of neural classifiers, a PCA (Principal Component Analysis) statistical method of reducing empirical data contained in the analyzed set was applied. The grain quality expressed by an optimal set of transformed descriptors was modelled using artificial neural networks (ANN). The input layer consisted of eight neurons with a linear Postsynaptic Function (PSP) and a linear activation function. The one hidden layer was composed of sigmoid neurons having a linear PSP function and a logistic activation function. One sigmoid neuron was the output of the network. The results obtained show that neural identification of digital images with application of Principal Component Analysis (PCA) combined with neural classification is an effective tool supporting the process of rapid and reliable quality assessment of BOJOS malting barley grains.

## 1. Introduction

Malting barley is a high-value crop with stringent requirements in terms of grain quality [1,2,3,4]. Between 6 and 10% of the barley grain yield is used for malt production for a variety of brewing, distilling and baking applications [5].

Brewing is one of the world’s largest markets. Interest in beer production continues to grow as evidenced by an explosion in scientific publications on improving beer production [3].

Malting barley cultivars are specific crops which are specially bred to achieve several specific qualitative and technological characteristics at the level of the grain. At present in Poland the number of registered malting barley cultivars is 32, including 29 spring cultivars and 3 winter cultivars [6]. The quality requirements of malting barley are related to processing efficiency and product quality in the malting and brewing industries. The presence of diseased or damaged grains is a strong negative factor [4,7]. Until now, quality assessment of grain has been usually performed by hand by malt-house-certified employees acting as experts who separate contaminants of malting barley and then calculate the percentage of contamination. Although very effective in terms of grain quality, this method is highly time-consuming and inefficient. Therefore, better and quicker methods are being sought for rapid estimation of contamination levels and seed damage in order to eliminate technologically unusable seeds. Hence, the need for new and improved methods and technologies to meet these demands. 

Computer vision has become one of the developing techniques used in agriculture for determination of grain quality and has been used to determine quality of the most relevant grains as maize, rice, wheat, soybean and barley [8,9,10,11,12,13,14,15,16]. The advantage of using this type of methods is the retained objectivity of the evaluation, increased speed and elimination of the subjective factor, such as expert fatigue, etc. [17,18].

In the case of malting barley, computer vision has been employed for identification of varieties [19,20,21,22] or to differentiate good from poor quality grains [14,15,16,23]. We have also made several attempts to introduce fast and low-cost methods based on image analysis and use of artificial neural networks [14,15,24,25]. This work is therefore a continuation of our previous studies where digital image analysis-based techniques with artificial neural networks (ANNs) were employed.

ANNs are nowadays one of the most dynamically developing branches of artificial intelligence [26,27,28,29,30]. A distinctive feature of ANNs, which has largely contributed to their practical application, is the ability of neural models to generalize information and perform parallel processing [30,31,32]. Properly performed neural network learning process allows the classification of new, previously unknown data, which increases the efficiency of the generated ANN. This capability allows the network to be applied where the problem cannot be solved in any other way [24,33,34,35,36,37,38,39,40]. Image analysis is a method that allows, for example, the extraction of interesting information from a set of acquired graphical data, encoded, for example, in the form of digital images. 

This study focuses on the identification of impurities and defects in malting barley (*Hordeum vulgare*) grains. The analysis of the problem area and consultations with numerous malting barley producers indicated that although several attempts have been made up to date, there is still a lack of an effective method for qualitative identification of barley grains, e.g., using information technology. 

The aim of the study was to develop an effective method for evaluation of the quality of BOJOS variety malting barley using information technology, with particular emphasis on modern artificial intelligence (AI) procedures. 

The paper proposes the use of neural image analysis methods, which are recognized (and used in practice) as identification instruments. An essential element of the process of neural quality assessment of grains presented in a graphic form is identification and extraction of the so-called representative parameters of grains (so-called graphic descriptors), representing information encoded in the form of digital images with use of a classical PCA statistical method. 

In addition to the scientific aspect, the studies carried out have a strong utilitarian thread. Among other things, they are dedicated as part of the structure of information systems (which are often expert systems) that support decision-making processes occurring during malting. This allows brewers to reduce their costs and increase automation in the initial phases of brewing activities.

## 2. Materials and Methods

### 2.1. Materials

Malting barley grains used for malt production were obtained from the Soufflet Polska malting plant in Poznań Poland). In this study, the currently purchased spring malting barley cultivar BOJOS was used. The examined seeds were characterized with the following technological parameters in 9° scale: synthetic index of brewing value—5.25, extractability—4, Wort viscosity—7 and Kolbach’s number—7 (according to the Soufflet Poland malting plant) [22].

### 2.2. Methods

#### 2.2.1. Preparation of Samples, Image Acquisition and Analysis

**Figure 1:**
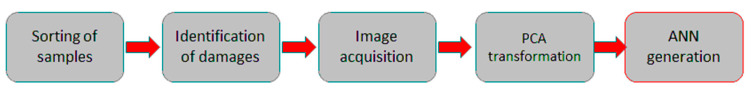
The scheme of the proposed procedures.

The flowchart of the proposed method is shown in Figure 1. 

**Figure 2:**
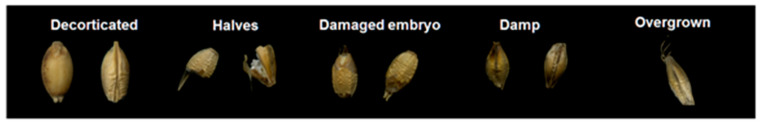
Examples of the types of BOJOS cultivar grain damages.

Samples of barley were prepared according to the following methodology (Polish Standard PN-R-74109):

- A total of 100 g of malting barley was weighted from a 1 kg sample using an electronic balance with an accuracy of 0.001 g,
- Weighted barley samples were sorted by means of a mechanical sorter Sortimat by grain size using 3 sieves of dimensions: 2.2 mm × 25 mm, 2.5 mm × 25 mm and 2.8 mm × 25 mm, and 3 fractions of samples were created: BOJOS 2.2 (218 grains), BOJOS 2.5 (866 grains), and BOJOS 2.8 (1386 grains),
- Basic types of grain damage of BOJOS variety were identified (examples on Figure 2),
- Percentage of damage/contamination in the samples with classification by type of contamination in 3 fractions of BOJOS barley cultivar was calculated (Table 1).

The above procedures allowed for the full description of damages and contaminants in the examined size fractions.

**Figure 3:**
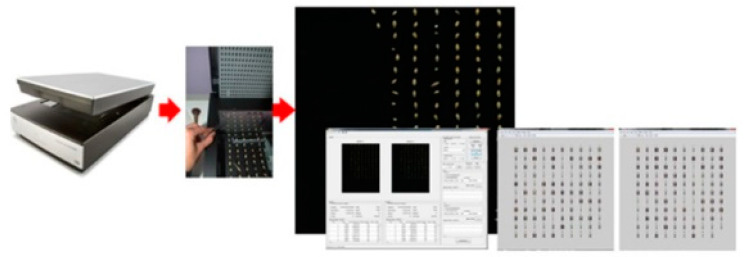
Acquisition and image processing of damaged malting barley grains using the original Hordeum v. 3.2 computer system created by B. Raba within MATLAB 2014b environment (MathWorks, Natick, MA, USA) using Image Processing Toolbox library.

Following the above steps, acquisition and analysis of images of BOJOS malting barley grains was executed (Figure 3) and a selection of 64 traits (considered representative) was made to describe malting barley grains as described in Table 1. The selected descriptors are: 12 geometric parameters (e.g., area. perimeter. etc.), 8 shape coefficients (e.g., Feret’s, Malinowska’s, etc.), 30 values of RGB and HSV color space models (e.g., maximum, minimum, median, mean, standard deviation. etc.) and 14 texture identifiers (e.g., matrices: event, band length distribution, image gradient, etc.) [15,25,41].

A large number of seeds’ descriptors (64) was obtained which can cause difficulties in the creation and operation of neural classifiers (such as an increase in the time to generate ANNs and the complexity of the classifier structure) [24,25,38,39,41]. For the above-mentioned reason, we decided to apply a statistical method of reducing the empirical data contained in the analyzed set, the Principal Component Analysis (PCA) technique, as an initial stage of developing a full computer vision procedure.

#### 2.2.2. Theoretical Background of the PCA Method

The PCA procedure is a widely used statistical method that aims to determine the so-called principal components, which are linear combinations of the observed (primary) variables [42]. Principal component analysis allows us to see which of the original data have the greatest influence on each component. The new components represent the raw data, which after the linear PCA transformation form a homogeneous and reduced group of input variables (descriptors) for ANN, being at the same time uncorrelated.

An important approach to solving the problem of compressing the dimension of the input signal vector is to reduce the dimension of the input space using an appropriate transformation. In such a transformation, the original set of variables is appropriately processed to create a new, smaller set of variables, but containing the maximum amount of information accumulated in the original set. 

If U denotes a p-dimensional random variable described by a vector $U^{T}=\left[U_{1},U_{2},\;\ldots ,\;U_{p}\right]$ then the density of a p-dimensional normal distribution can be defined as below [43].

(1) $$f_{µ,\sum }\left(U\right)=\frac{1}{{\left(2\pi \right)}^{\frac{p}{2}}{\left|\sum \right|}^{\frac{1}{2}}}\;\exp\left[-\frac{1}{2}\;{\left(U-µ\right)}^{T\;}\sum^{-1}\left(U-µ\right)\right]$$

where:

$µ=E\left(U\right)=\left[E\left(U_{1}\right),\;E\left(U_{2}\right),\;\ldots ,\;E\left(U_{p}\right)\right]$ is a vector of expected values of the variable U, while the integral operator *E*(…) is expressed as (e.g., for the *U*_1_ component): $E\left[U_{1}\right]=\int_{-\infty }^{+\infty }u_{1}dF$ where *F*(*u*) is the distribution of the random variable *U*_1_ (integration is performed in the Lebesque–Stieltjes sense).

(2) $$\sum =D\left(U\right)=\left[\begin{array}{cccc} \mathrm{var}\;\left(U_{1}\right) & \mathrm{cov}\;\left(U_{1}U_{2}\right) & \ldots & \mathrm{cov}\;\left(U_{1}U_{p}\right)\\ \mathrm{cov}\;\left(U_{2}U_{1}\right) & \mathrm{var}\;\left(U_{2}\right) & \ldots & \mathrm{cov}\;\left(U_{2}U_{p}\right)\\ \ldots & \ldots & \ldots & \ldots \\ \mathrm{cov}\;\left(U_{p}U_{1}\right) & \mathrm{cov}\;\left(U_{p}U_{2}\right) & \ldots & \mathrm{var}\;\left(U_{p}\right) \end{array}\right]\;\mathrm{is}\;a\;\mathrm{symmetric}\;\mathrm{covariance}\;\mathrm{matrix},$$

where:

var (*U_p_*) is the variance of a random variable *U_p_*, and

cov (*U_i_U_p_*) is the covariance of the variables *U_i_* and *U_p_*.

The covariance matrix is a symmetric (square), real and non-personal matrix. 

If $\lambda_{1}\geq \lambda_{2}\geq \ldots \geq \lambda_{p}$ are roots of the characteristic equation of matrix (2), then they are also eigenvalues of the **∑** matrix, while P_1_, P_2_…P_p_ are the corresponding eigenvectors of this matrix. Then by performing elementary transformations as follows [42,43]:

(3) $$\sum =\lambda_{1}\;P_{1}P_{1}^{T}+\lambda_{2}P_{2}P_{2}^{T}+\cdots +\lambda_{p}P_{p}P_{p}^{T}\;I=P_{1}P_{1}^{T}+P_{2}P_{2}^{T}+\cdots +P_{p}P_{p}^{T}$$

and

(4) $$\;P_{i}^{T}P_{i}=\lambda_{i},P_{j}^{T}P_{j}=0\;\mathrm{for}\;i\text{≠}j\;$$

Introducing new random variables: $Y_{i}=P_{i}^{T\;}U\;$ and based on Equation (3), the following relations can be obtained:

(5) $$\;P_{i}^{T}\sum P_{i}=\lambda_{i},P_{j}^{T}P_{j}=0\;\mathrm{for}\;i\neq j\;\;\mathrm{cov}\;\left(P_{i}^{T}U,\;P_{j}^{T}U\right)=P_{i}^{T}P_{j}=0\;\;\;\mathrm{for}\;i\neq j$$

By transforming the covariance matrix (2), a spectral matrix is produced which enables us to determine the principal directions (components), e.g., by identifying the roots of the characteristic equation of the matrix **Σ**:

(6) $$\sum =\left[\begin{array}{cccc} \mathrm{var}\;\left(U_{1}\right) & \mathrm{cov}\;\left(U_{1}U_{2}\right) & \ldots & \mathrm{cov}\;\left(U_{1}U_{p}\right)\\ \mathrm{cov}\;\left(U_{2}U_{1}\right) & \mathrm{var}\;\left(U_{2}\right) & \ldots & \mathrm{cov}\;\left(U_{2}U_{p}\right)\\ \ldots & \ldots & \ldots & \ldots \\ \mathrm{cov}\;\left(U_{p}U_{1}\right) & \mathrm{cov}\;\left(U_{p}U_{2}\right) & \ldots & \mathrm{var}\;\left(U_{p}\right) \end{array}\right]\;\to \;\to \hat{\sum }=\left[\begin{array}{cccc} \lambda_{1} & 0 & \ldots & 0\\ 0 & \lambda_{2} & \ldots & 0\\ \ldots & \ldots & \ldots & \ldots \\ 0 & 0 & \ldots & \lambda_{p} \end{array}\right]=\left[\begin{array}{cccc} \mathrm{var}{\left(U_{1}\right)}_{max_{1}} & 0 & \ldots & 0\\ 0 & \mathrm{var}{\left(U_{2}\right)}_{max_{2}} & \ldots & 0\\ \ldots & \ldots & \ldots & \ldots \\ 0 & 0 & \ldots & \mathrm{var}{\left(U_{p}\right)}_{max_{p}} \end{array}\right]\;$$

where:

(7) $$\mathrm{var}\;{\left(U_{1}\right)}_{max_{1}}>\mathrm{var}\;{\left(U_{2}\right)}_{max_{2}}>\cdots >\mathrm{var}\;{\left(U_{p}\right)}_{max_{p}}$$

Relationship (5) implies that an orthogonal transformation of the form **Y** = **P**^T^**U** converts the correlated random (primary) variables into the reduced uncorrelated variables. These are defined to maximize the variation that is not explained by the previous component. The variables in the input file with the largest variance have a dominant influence on the outcome, which is important if the variables represent comparable quantities [42,43]. 

## 3. Results and Discussion

The paper focuses on the problem of reducing the number of characteristic parameters of digital images presenting damage of malting barley grains of three size fractions. 

In our experiment, malting barley seeds were divided into three size groups (2.2, 2.5 and 2.8 mm). According to the research, seed size and external characteristics affect malt quality due to differences in protein, starch and enzyme content. The extract potential of grains is probably determined by a combination of the parameters such as grain dimensions, ratios of these dimensions, grain shape, uniformity of these within a sample and also surface textures [4,44]. Smaller grains generally have lower starch and higher protein levels which reduces the extract potential. Large grains generally have increased levels of starch and therefore more extract potential [2]. Based on the above, other authors also separated barley grain samples into similar grain size fractions: 2.8–2.5 mm, 2.5–2.2 mm and <2.2 mm [45,46].

Visual assessment of malting barley quality is the first step in the malting process. All of the tested seeds were fully mature. As can be seen from Table 1, as the size of the seeds increased, the number of good quality grains increased, and at the same time the number of mold-infected grains decreased. A decrease in the number of dehulled seeds was also observed. The highest proportion of dehulled seeds was in fraction 2.5, while the highest number of seeds with dark ends and affected by pests was observed for fraction 2.2. Fraction 2.5 had the highest proportion of seeds with the embryo killed. A small contribution of sprouted grains was observed for the larger seeds. Small amounts of admixtures of other seeds were found in seeds from fractions characterized by smaller size. 

In order to automate the process of evaluating the quality of the seeds, we used computer vision where the recorded image features of the seeds were analysed. 

The identification of healthy, high quality barley grains requires an adequate classifier. We have chosen to perform a selection from the several parameters extracted from seed images (Table 2). A total of 12 geometric parameters, 8 shape factors, 30 values characterising colour space models (15 for RGB and 15 for HSV) as well as 14 texture descriptors were extracted. We have applied this procedure previously to analyse the images of other agricultural products [6,14,15,16,18,24,25,27,28,29,30,35,36,38,39,41].

It was assumed that it is possible to estimate the optimal set of representative features of digital images of malting barley grains within size groups, necessary in the process of generating a neural classifier. We have attempted to describe the quality of malting barley of other varieties based on digital images in the previous studies [6,15,24,25], but in the work presented here, we applied dimension reduction of descriptors in the initial phase of image analysis for the first time using the classical PCA statistical method.

### 3.1. Reduction of the Number of Descriptors Using the PCA Approach

Principal component analysis (PCA) can be used to discover regularities between variables (descriptors). The components obtained by this method are a linear combination of the studied input variables (primary descriptors). PCA facilitates identification of those initial variables that have the greatest influence on the appearance of individual principal components, i.e., those that form a homogeneous group [47,48,49,50,51,52,53]. The principal component (in which the variance is maximal) is then the representative of this group.

On the basis of variance values explained by successive principal components, eight first components were identified (Kaiser criterion) for the examined three size fractions of malting barley of BOJOS variety. The explained variance values for the these components are shown in Table 3.

Parameters significant for the quality evaluation of malting barley of the BOJOS cultivar (out of 64 descriptors classified into 4 groups (geometric parameters, shape factors, values of colour space models or texture), shown in Table 2) proved to be the best descriptors (Table 3). Colour features of kernels included mean, variance, and ranges of the red^®^, green (G) and blue (B) colour primaries and the derived hue (H), saturation (S) and (V) value. Among texture parameters GLCM provides information about the distribution of grey level intensities with respect to the relative position of the pixels with equal intensities.

The most important graphical descriptors for three size fractions (2.2; 2.5; 2.8) of the BOJOS cultivar are shown in the order of significance level of assignment to the first principal component in Table 4.

Descriptors “Circumference” (perimeter), “MinorAxisLength” (width, plumpness), “G Min” (for RGB colour model: green colour minimum) and “V Min” (for HSV color model: brightness minimum) were found as dominant parameters in all three fractions of the BOJOS variety. Thus, it can be concluded that the graphical information encoded in the form of selected geometric parameters and colour space models is representative of all three fractions. Interestingly, shape factors were non-relevant.

PCA is the most common and popular linear dimension reduction approach [47]. It has been used for years because of its conceptual simplicity and computation efficiency. It is a practical application of the technique of finding eigenvalues and eigenvectors of a square matrix. It is a linear transformation that determines the directions of maximum variation of the original input data as it rotates the coordinate system in such a way that the maximum dispersion (variance) of the data occurs (after the transformation) along the new axes. It retains as much valuable information as possible in the transformed data. The directions of maximum variance are not necessarily the directions of maximum information. PCA is often used to reduce the size of a statistical dataset by discarding the last factors. It is also possible to look for a substantive interpretation of the factors (depending on the type of data) which allows for better understanding of the nature and essence of the empirical data being analyzed.

The PCA approach is applied in many areas such as noise reduction, pattern recognition, regression estimation, and image recognition or generally in reduction of dimension of a digital image. In signal processing, PCA is often used to compress the input signal [48,49,50,51,52,53].

Concerning barley grain images, PCA has been previously applied in pre-processing of the data used in its varietal classification by morphological features [54] or in remote sensing and barley crop classification [55]. PCA was also used in analysis of the images of barley leaves to detect infection with *Magnaporthe oryzae* [56].

Nielsen at al. (2003) [57] used PCA analysis for evaluation of micro-malted spring and winter barley quality expressed by different chemical and malting parameters evaluated according to official methods of the European Brewery Convention.

There are no publications in the literature on the application of classical PCA analysis to reduce the number of input parameters for neural modelling extracted from image processing of brewing barley.

### 3.2. Artificial Neural Networks Modelling

A set of 30 multilayer perceptron neural topologies was generated for each fraction [12,36]. The optimal networks turned out to be topologies with the following structures: MLP: 8-14-1 (fraction 2.2), MLP 8-19-1 (fraction 2.5) and MLP 8-8-1 (fraction 2.8). The input layers consisted of eight neurons with a linear PSP (Postsynaptic Function) and a linear activation function. The one hidden layer was composed of sigmoid neurons having a linear PSP function and a logistic activation function. One sigmoid neuron was the output of the network. It included one nominal two-state variable (good grain, damaged grain).

The generated neural models were thought by the method of BP (Back Propagation) in 10 cycles of 1000 epochs and then optimized with the CG (Conjugate Gradients) algorithm for 600 epochs. The following parameters were adopted in the learning process with the BP error back propagation algorithm [31,41]:

- Decreasing learning coefficient: from η = 0.2 to η = 0.1,
- Momentum factor: α = 0.4.

ANNs were optimized by searching for the right set of synaptic weights that form connections between neurons of neighbouring network layers. As a criterion for optimization, a set of weights was taken, which (implemented in the network) guaranteed the minimum of error that the ANN generates during its operation. For this purpose, an appropriate algorithm was selected and used to minimize (optimize) the error function of the network. In this work, the standard RMS (Root Mean Square) error function (8) was applied which is a hyper paraboloid. This procedure is commonly used in the ANN optimization process regardless of the choice of ANN simulator [30,31].

(8) $$\mathrm{RMS}=\sqrt{\frac{\sum_{i=1}^{n}{\left(y_{1}-z_{1}\right)}^{2}}{n}}$$

where:

*n* is the number of cases,

*y_i_* is the real values, and

*z_i_* is the values determined using the network.

The structure of the training file is important in the process of generating the neural classifier. Minimizing the dimension of the descriptors vector, which is the input variables of the created neural model, allowed for the use of the empirical data in a reduced form. An effective neural models supporting decision-making processes in the production of malting barley was proposed on the example of the popular BOJOS variety. 

The RMS error for MLP: 8-14-1, MLP: 8-19-1 and MLP: 8-8-1 is presented in Table 5 The RMS error is usually the most convenient value for interpretation to describe the total error of the network. In our case, the quality of the generated neural network can be considered very satisfactory. 

The low and very similar value of the RMS error for the training, validation and test set indicates good generalization properties of the generated ANN. Its small value in turn implies very good classification properties of the generated models.

The neural topologies generated on the basis of training sets containing eight input variables (significant graphic descriptors) created for three size fractions (samples: 2.2, 2.5 and 2.8) of the BOJOS variety, are presented in Table 6.

The generated MLP (Multilayer Perceptron) topologies were proved optimal (for each fraction). 

MLP-type unidirectional neural networks are among the best studied and most widely used network topologies in practice. MLP multilayer perceptron represents the so-called class of parametric neural models. The created MLP networks are one-way networks. They are taught using the “with the teacher” technique (i.e., algorithms modify weights and threshold values using training files containing both input values and set output values). They have a multi-layer architecture. There is an input layer, a hidden layer and an output layer. Connections allow exclusively for communication between neurons in adjacent layers. Neurons constituting the network aggregate the input data by determining the sum of the weighted inputs (using the linear aggregation formula). The activation function of input neurons is linear, hidden neurons non-linear, and output neurons are generally non-linear. 

In our work, a set of 64 features representative of digital images of malting barley grains of the BOJOS variety was extracted (Table 1). A large number of descriptors (especially in relation to the number of learning cases—176) negatively affected both the process of creation and exploitation of neural classifiers as it prolonged the ANN generation process and significantly increased the complexity of the classifier structure. This, in turn, hindered the implementation of the generated neural classifier in the created information system. Taking the above into account, the empirical data were reduced with PCA, which resulted primarily in reducing the size of the generated ANNs. In the stochastic sense, the results generated by the “large” ANN (without PCA) and with the “reduced” ANNs are similar.

Generally, the problem of identification of damaged malting barley grains is not so frequently reported. Computer vision supported by neural networks has been most frequently applied to identify varieties of different grains, including barley [19,20,21,22,23,25,54,58]. Neural image analysis has been used to examine corn and barley kernel damages [6] or the mechanical damages in grains [24]. Kozłowski and Szczypiński used convolutional neural networks for identification of barley grain defects [58], while Kociołek et al. (2017) [59] reported image preprocessing steps of barley grain inspection system. The main preprocessing steps were: segmentation of grain kernel images, identification of dorsal and ventral sides of the kernels as well as aligning them with respect to the germ-brush direction.

This paper is one of the few research reports dealing strictly with the identification of contamination and damage to malting barley. Earlier studies on identification of damaged malting barley grains from our research group were focused on artificial neural networks used only to classify grain damage images of three selected spring varieties: Beatrix, Sebastian and Xanadu [14]. In the following work, we applied a hybrid method on image features of Sebastian variety in which auto-associative artificial neural networks were used to reduce the dimension of the input vector. Networks of this type can be used successfully, mostly to reduce the dimension of the vector representing the input data, which significantly supports the process of creation of an optimal neural topology to solve a given problem [15].

In the work presented here, the classical PCA method was applied for dimension reduction and the disposal of correlated variables prior to neural modelling.

## 4. Conclusions

The use of neural modelling and image analysis methods for the identification of malting barley quality in the example of BOJOS variety proved to be an appropriate method that could support decision-making processes in the brewing process. Reduction of the number of graphical descriptors allowed for graphical identification of defects using small neural networks of the multilayer perceptron type (MLP). This showed that few of the identified representative parameters (graphical descriptors) are sufficient for proper quality classification of malting barley. The results obtained show that neural identification of digital images using a classical PCA analysis is an effective tool supporting the process of rapid and reliable quality assessment of malting barley grains of the BOJOS variety. Due to a small number of obtained images of grains (176) in relation to the number of primary descriptors (64), it was appropriate to use the PCA method to reduce the dimension of the input replacement vector. The parameters most important in the process of the quality assessment of malting barley of the BOJOS variety were identified. Reduced descriptors, two geometric and two describing color space models (RGB and HSV), showed to be the dominant characteristic parameters for digital images of all size fractions of the BOJOS variety. Qualitative analysis of the reduced neural models for individual seed fractions of the BOJOS variety showed that the best classification abilities were achieved by neural topologies (MLP: 8-14-1, MLP: 8-19-1, MLP: 8-8-1) generated on the basis of compressed training files. 

The conducted research indicated the usefulness of the developed method as an instrument effectively supporting decision-making processes occurring during beer production.

## Figures and Tables

**Table 1:** Contaminant content in the examined size fractions of malting barley of BOJOS variety [%].

Characteristics		Size Fraction	
	2.2	2.5	2.8
No pollution/Good quality grain	40.27	48.04	57.09
Mold-infected grain	49.54	40.88	35.57
Halves	4.47	2.54	0.94
Grain partially/completely dehulled	1.83	6.35	3.84
“Rainy weather” (with dark ends)	3.31	1.27	2.28
Grain with embryo killed	0	0.69	0.25
Sprouted grain	0	0	0.03
Grain affected by pests	0.46	0	0
Other grains/seeds	0.12	0.23	0

**Table 2:** Parameters extracted from malting barley seeds’ images using a Hordeum v.3.2 computer system (number of parameters).

Geometric Parameters (12)	Shape Factors (8)	Values of Colour Space Models (15) (RGB) (15) (HSV)		Texture (14)
‘Area’. ‘Circumference’. ‘Max. height’. ‘Max. width’. ‘Rmax’. ‘Rmin’. ‘OAR’. ‘Circle diameter’. ‘Eccentricity’. ‘Ellipse’. ‘MajorAxisLength’. ‘MinorAxisLength’. ‘Solidity’.	‘C.Feret’. ‘C.Malinowska’. ‘C.Circularity#1’. ‘C.Circularity#2’. ‘C.Ellipsicity’. ‘C.Blair-Bliss’. ‘C.Haralick’. ‘C.Danielsson’.	‘R Max’. ‘R Min’. ‘R Mean’. ‘R Median’. ‘R STD’. ‘G Max’. ‘G Min’. ‘G Mean’. ‘G Median’. ‘G STD’. ‘B Max’. ‘B Min’. ‘B Mean’. ‘B Median’. ‘B STD’.	‘H Max’. ‘H Min’. ‘H Mean’. ‘H Median’. ‘H STD’. ‘S Max’. ‘S Min’. ‘S Mean’. ‘S Median’. ‘S STD’. ‘V Max’. ‘V Min’. ‘V Mean’. ‘V Median’. ‘V STD	‘MGmean’. ‘MGvar’. ‘MGskew’. ‘MGkurto’. ‘ZeroPercent’. ‘rSRE’. ‘rLRE’. ‘rRLN’. ‘rFIR’. ‘rGLN’. ‘GLCMContrast’. ‘GLCMCorrelation’. ‘GLCMEnergy’. ‘GLCMHomogeneity’.

# parameter number.

**Table 3:** Values of variances explained for 8 consecutive principal components.

	Size Fraction		
	2.2	2.5	2.8
No. main component	Variance [%]		
1	22.03	21.59	22.97
2	18.54	18.10	17.20
3	15.88	15.34	13.59
4	8.69	8.30	8.26
5	6.80	6.85	7.40
6	4.35	4.94	5.95
7	3.63	4.10	3.77
8	3.32	3.08	3.06

**Table 4:** Graphical descriptors for 3 fractions (2.2, 2.5, 2.8) of BOJOS variety samples (order by significance level of assignment to the first principal component).

Fraction	Eight of the Most Important Primary Graphic Descriptors
2.2	Circumference, GLCMCorrelation, GLCM Homogeneity, R Median, MinorAxisLength, G Min, S Max, V Min
2.5	Circumference, GLCMContrast, S Mean, MinorAxisLength, S Max, S STD, G Min, V Min
2.8	Circumference, V Mean, Mgmean, S Median, MinorAxisLength, S STD, G Min, V Min

**Table 5:** RMS error for generated MLP: 8-14-1, MLP: 8-19-1 and MLP: 8-8-1 neural topologies.

	Model		
RMS Error *	MLP: 8-14-1	MLP: 8-19-1	MLP: 8-8-1
RMS (training file)	0.017233	0.010752	0.010869
RMS (testing file)	0.012034	0.0105261	0.010416
RMS (validation file)	0.011213	0.0108675	0.010638

* dimensionless quantity.

**Table 6:** Best neural network models for each fraction of the BOJOS variety samples.

		BOJOS		
	Sample	2.2	2.5	2.8
ANN Quality				
Statistical v. 10 (StatSoft Polska, Cracov, Poland)		MLP: 8-14-1	MLP: 8-19-1	MLP: 8-8-1
ANN structure		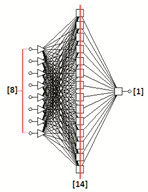	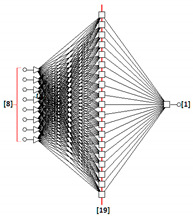	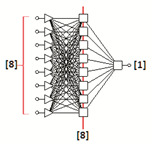
Quality of the training file [%]		85.40	92.43	91.70
Quality of the testing file [%]		83.13	94.74	95.87
Quality of the validation file [%]		82.75	92.89	94.36
Learning algorithms used		BPCG—1600 epochs	BPCG—1600 epochs	BPCG—1600 epochs

Where: BP—back propagation method; CG—conjugate gradient method.

## Data Availability

Data available on request.

## References

[1] Nogueira M.S., Decundo J., Martinez M., Dieguez S.N., Moreyra F., Moreno M.V., Stenglein S.A. (2018). Natural Contamination with Mycotoxins Produced by *Fusarium graminearum* and *Fusarium poae* in Malting Barley in Argentina. Toxins.

[2] Kumar D., Kumar V., Verma R.P.S., Kharub A.S., Sharma I. (2013). Quality Parameter Requirement and Standards for Malt Barley—A Review. Agric. Rev..

[3] Pallottino F., Cimini A., Costa C., Antonucci F., Menesatti P., Moresi M. (2020). Bibliometric analysis and mapping of publications on brewing science from 1940 to 2018. J. Inst. Brew..

[4] Rani H., Bharadwaj R.D. (2021). Quality attributes for barley malt: “The backbone of beer”. J. Food Sci..

[5] Tricase C., Amicarelli V., Lamonaca E., Rana R.L., Tabele Z. (2018). Economic analysis of the barley market and related uses. Grasses as Food and Feed, 10.

[6] Nowakowski K., Boniecki P., Tomczak R.Ł., Raba B. Identification process of corn and barley kernels damages using neural image analysis. Proceedings of the Third International Conference on Digital Image Processing (ICDIP 2011).

[7] Beccari G., Prodi A., Tini F., Bonciarelli U., Onofri A., Oueslati S., Limayma M., Covarelli L. (2017). Changes in the Fusarium Head Blight Complex of Malting Barley in a Three-Year Field Experiment in Italy. Toxins.

[8] Arngren M., Hansen P.W., Eriksen B., Larsen J., Larsen R. (2011). Analysis of Pregerminated Barley Using Hyperspherical Image Analysis. Agric. Food Chem..

[9] Aznan A., Gonzalez Viejo C., Pang A., Fuentes S. (2021). Computer Vision and Machine LearningAnalysis of Commercial Rice Grains:A Potential Digital Approach forConsumer Perception Studies. Sensors.

[10] He X., Zhao T., Shen F., Liu Q., Fang Y., Hu Q. (2021). Online detection of naturally DON contaminated wheat grains from China using Vis-NIR spectroscopy and computer vision. Biosyst. Eng..

[11] Velesaca H.O., Suárez P.L., Mira R., Sappa A.D. (2021). Computer vision based food grain classification: A comprehensive survey. Comput. Electron. Agric..

[12] Xu P., Yang R., Zeng T., Zhang J., Zhang Y., Tan Q. (2021). Varietal classification of maize seeds using computer vision and machine learning techniques. J. Food Process. Eng..

[13] de Medeiros A.D., Capobiango N.P., da Silva J.M., da Silva L.J., da Silva C.B., dos Santos Dias D.C.F. (2020). Interactive machine learning for soybean seed and seedling quality classification. Sci. Rep..

[14] Pilarska A.A., Boniecki P., Idzior-Haufa M., Maciej Zaborowicz M., Pilarski K., Przybylak A., Piekarska-Boniecka H. (2021). Image Analysis Methods in Classifying Selected Malting Barley Varieties by Neural Modelling. Agriculture.

[15] Boniecki P., Raba B., Pilarska A.A., Sujak A., Zaborowicz M., Pilarski K., Wojcieszak D. (2021). Neural Reduction of Image Data in Order to Determine the Quality of Malting Barley. Sensors.

[16] Stejskal V., Vendl T., Li Z., Aulicky R. (2020). Efficacy of visual evaluation of insect-damaged kernels of malting barley by *Sitophilus granarius* from various observation perspectives. J. Stored Prod. Res..

[17] Przybył K., Zaborowicz M., Koszela K., Boniecki P., Mueller W., Raba B., Lewicki A. Organoleptic Damage Classification of Potatoes with the Use of Image Analysis in Production Process. Proceedings of the Sixth International Conference on Digital Image Processing (ICDIP 2014).

[18] Janczak D., Lewicki A., Mazur R., Boniecki P., Dach J., Przybył J., Pawlak M., Pilarski K., Czekala W. The Selected Examples of the Application of Computer Image Analysis in the Assessment of Environmental Quality. Proceedings of the Fifth International Conference on Digital Image Processing (ICDIP 2013).

[19] Szczypiński P.M., Klepaczko A., Zapotoczny P. (2015). Identifying barley varieties by computer vision. Comput. Electron. Agric..

[20] Kozłowski M., Górecki P., Szczypiński P.M. (2019). Varietal classification of barley by convolutional neural networks. Biosyst. Eng..

[21] Shi Y., Patel Y., Rostami B., Chen H., Wu L., Yu Z., Li Y. (2022). Barley Variety Identification by iPhone Images and Deep Learning. J. Amer. Soc. Brew. Chem..

[22] Shah S.A.A., Luo H., Pickupana P.D., Ekeze A., Sohel F., Laga H., Li C., Paynter B., Wang P. (2022). Automatic and fast classification of barley grains from images: A deep learning approach. Smart Agric. Technol..

[23] Ramirez-Paredes J.-P., Hernandez-Belmonte U.-H. (2020). Visual quality assessment of malting barley using color, shape and texture descriptors. Comp. Electron. Agric..

[24] Nowakowski K., Boniecki P., Dach J. The Identification Of Mechanical Damages Of Kernels. Basis Of Neural Image Analysis. Proceedings of the 2009 International Conference on Digital Image Processing.

[25] Nowakowski K., Boniecki P., Tomczak R.J., Kujawa S., Raba B. Identification of malting barley varieties using computer image analysis and artificial neural networks. Proceedings of the Fourth International Conference on Digital Image Processing (ICDIP 2012).

[26] Iosa M., Benedetti M.G., Antonucci G., Paolucci S., Morone G. (2022). Artificial Neural Network Detects Hip Muscle Forces as Determinant for HarmonicWalking in People after Stroke. Sensors.

[27] Zaborowicz M., Boniecki P., Koszela K., Przybyl J., Mazur R., Kujawa S., Pilarski K. Use of Artificial Neural Networks in the Identification and Classification of Tomatoes. Proceedings of the Fifth International Conference on Digital Image Processing (ICDIP 2013).

[28] Dach J., Czekała W., Boniecki P., Lewicki A., Piechota T. Specialised internet tool for biogas plant modelling and marked analysing. Proceedings of the 2nd International Conference on Manufacturing and Applied Research.

[29] Boniecki P., Dach J., Nowakowski K., Jakubek A. Neural image analysis of maturity stage during composting of sewage sludge. Proceedings of the International Conference on Digital Image Processing.

[30] Boniecki P. (2008). Elements of Neural Modeling in Agriculture.

[31] Fausett L. (1994). Fundamentals of Neural Networks.

[32] Bishop C. (1995). Neural Networks for Pattern Recognition.

[33] Pavićević M., Popović T. (2022). Forecasting Day-Ahead Electricity Metrics with Artificial Neural Networks. Sensors.

[34] Salehuddin N.F., Omar M.B., Ibrahim R., Bingi K. (2022). A Neural Network-Based Model for Predicting Saybolt Color of Petroleum Products. Sensors.

[35] Boniecki P., Nowakowski K., Tomczak R., Kujawa S., Piekarska-Boniecka H. The application of the Kohonen neural network in the non-parametric quality-based classification of tomatoes. Proceedings of the Fourth International Conference on Digital Image Processing (ICDIP 2012).

[36] Boniecki P., Piekarska-Boniecka H., Koszela K., Nowakowski K., Kujawa S., Majewski A., Weres J. (2015). Raba, B. Neural identification of selected apple pests. Comput. Electron. Agric..

[37] Deng F., Li S.-Q., Zhang X.-R., Zhao L., Huang J.-B., Zhou C. (2022). An Intelligence Method for Recognizing Multiple Defects in Rail. Sensors.

[38] Przybylak A., Boniecki P., Koszela K., Ludwiczak A., Zaborowicz M., Lisiak D., Stanisz M., Slosarz P. (2016). Estimation of intramuscular level of marbling among Whiteheaded Mutton Sheep lambs. J. Food Eng..

[39] Zaborowicz M., Fojud A., Koszela K., Mueller W., Górna K., Okoń P., Piekarska-Boniecka H. Dedicated computer system AOTK for image processing and analysis of horse navicular bone. Proceedings of the Ninth International Conference on Digital Image Processing (ICDIP 2017).

[40] Sujak A., Jakubas D., Kitowski I., Zbyryt A., Bzoma S., Boniecki P. (2022). Identification of factors affecting environment contamination represented by post-hatching eggshells of a common colonial waterbird with usage of artificial neural networks. Sensors.

[41] Boniecki P., Koszela K., Piekarska-Boniecka H., Nowakowski K., Przybyl J., Zaborowicz M., Raba B., Dach J. Identification of Selected Apple Pests Based on Selected Graphical Parameters. Proceedings of the Fifth International Conference on Digital Image Processing (ICDIP 2013).

[42] Rao C.R. (1982). Linear Models of Mathematical Statistics.

[43] Ahmad Z., Nguyen T.-H., Ahmad S., Nguyen C.D., Kim J.-M. (2022). Multistage Centrifugal Pump Fault Diagnosis Using Informative Ratio Principal Component Analysis. Sensors.

[44] Hoyle A., Brennan M., Pitts N., Jackson G.E., Hoad S. (2020). Relationship between specific weight of spring barley and malt quality. J. Cereal Sci..

[45] Magliano P.N., Prystupa P., Gutiérrez-Boem F.H. (2014). Protein content of grains of different size fractions in malting barley. J. Inst. Brew..

[46] Yu W., Tan X., Zou W., Hu Z., Fox G.P., Gidley M.J., Gilbert R.G. (2017). Relationships between Protein Content, Starch Molecular Structure and Grain Size in Barley. Carbohydr. Polym..

[47] Postma L.J.E.O., van den Herik H.J., van der Maaten L.J. (2009). Dimensionality reduction: A comparative review. J. Mach. Learn. Res..

[48] Cheng D., Zhao D., Zhang J., Wei C., Tian D. (2021). PCA-Based Denoising Algorithm for Outdoor Lidar Point Cloud Data. Sensors.

[49] Ng S.C. (2017). Principal component analysis to reduce dimension on digital image. Procedia Comput. Sci..

[50] Song L., Ma H., Wu M., Zhou Z., Fu M., Peng Y., Yu K., Lu J., Jiang X. (2018). A Brief Survey of Dimension Reduction. Proceedings of the International Conference on Intelligent Science and Big Data Engineering.

[51] Xia Z., Chen Y., Xu C. (2021). Multiview PCA: A Methodology of Feature Extraction and Dimension Reduction for High-Order Data. IEEE Trans. Cybern..

[52] Uddin P., Mamun A., Hossain A. (2021). PCA-based Feature Reduction for Hyperspectral Remote Sensing Image Classification. IETE Techn. Rev..

[53] Ji M., Yuyu Y. (2019). Dimension reduction of image deep feature using PCA. J.Vis. Commun. Image Represent..

[54] Zapotoczny P., Zielinska M., Nita Z. (2008). Application of image analysis for the varietal classification of barley: Morphological features. J. Cereal Sci..

[55] Guo J., Li H., Ning J., Han W., Zhang W., Zhou Z.-S. (2020). Feature Dimension Reduction Using Stacked Sparse Auto-Encoders for Crop Classification with Multi-Temporal, Quad-Pol SAR Data. Remote Sens..

[56] Zhou R.-Q., Jin J.-J., Li Q.-M., Su Z.-Z., Yu X.-J., Tang Y., Luo S.-M., He Y., Li X.-L. (2019). Early Detection of *Magnaporthe oryzae*-Infected Barley Leaves and Lesion Visualization Based on Hyperspectral Imaging. Front. Plant Sci..

[57] Nielsen J.P., Munck L. (2003). Evaluation of malting barley quality using explanatory data analysis. I. Extraction of information from micro-malting data of spring and winter barley. J. Cereal Sci..

[58] Kozłowski M., Szczypiński P.M. (2019). Barley defects identification by convolutional neural networks. Proceedings of the International Conference on Information Technologies in Biomedicine.

[59] Kociołek M., Szczypiński P.M., Klepaczko A. Preprocessing of barley grain images for defect identification. Proceedings of the 2017 Signal Processing: Algorithms, Architectures, Arrangements, and Applications (SPA).

